# A TolC-Like Protein of *Actinobacillus pleuropneumoniae* Is Involved in Antibiotic Resistance and Biofilm Formation

**DOI:** 10.3389/fmicb.2016.01618

**Published:** 2016-10-24

**Authors:** Ying Li, Sanjie Cao, Luhua Zhang, Gee W. Lau, Yiping Wen, Rui Wu, Qin Zhao, Xiaobo Huang, Qigui Yan, Yong Huang, Xintian Wen

**Affiliations:** ^1^Research Center of Swine Diseases, College of Veterinary Medicine, Sichuan Agricultural UniversityChengdu, China; ^2^Department of Pathobiology, University of Illinois at Urbana-ChampaignUrbana, IL, USA

**Keywords:** TolC, biofilm formation, multidrug resistance, PAβN, *Actinobacillus pleuropneumoniae*

## Abstract

*Actinobacillus pleuropneumoniae* is the etiologic agent of porcine contagious pleuropneumonia, a significant disease that causes serious economic losses to the swine industry worldwide. Persistent infections caused by bacterial biofilms are recalcitrant to treat because of the particular drug resistance of biofilm-dwelling cells. TolC, a key component of multidrug eﬄux pumps, are responsible for multidrug resistance (MDR) in many Gram-negative bacteria. In this study, we identified two TolC-like proteins, TolC1 and TolC2, in *A. pleuropneumoniae.* Deletion of *tolC1*, but not *tolC2*, caused a significant reduction in biofilm formation, as well as increased drug sensitivity of both planktonic and biofilm cells. The genetic-complementation of the *tolC1* mutation restored the competent biofilm and drug resistance. Besides, biofilm formation was inhibited and drug sensitivity was increased by the addition of phenylalanine-arginine beta-naphthylamide (PAβN), a well-known eﬄux pump inhibitor (EPI), suggesting a role for EPI in antibacterial strategies toward drug tolerance of *A. pleuropneumoniae*. Taken together, TolC1 is required for biofilm formation and is a part of the MDR machinery of both planktonic and biofilm cells, which could supplement therapeutic strategies for resistant bacteria and biofilm-related infections of *A. pleuropneumoniae* clinical isolate SC1516.

## Introduction

*Actinobacillus pleuropneumoniae*, a facultative anaerobic Gram-negative coccobacillus belonging to the family *Pasteurellaceae*, is the etiological agent of porcine pleuropneumonia characterized by serious fibrino-hemorrhagic necrotizing pneumonia and fibrinous pleurisy (Bosse et al., 2002). This disease often manifests as severe outbreaks, causing significant economic losses in porcine industry worldwide. Even though considerable progress has been made in unmasking the pathogenesis of *A. pleuropneumoniae*, much remains to be studied (Chiers et al., 2010). In particular, little is known regarding how *A. pleuropneumoniae* confers resistance against bactericidal substances for survival and spread within the host.

Bacteria often live in compact microbial communities enclosed in a self-produced matrix called biofilms that attach to biotic or abiotic surfaces (Van Acker et al., 2014). Biofilms protect bacterial cells by decreasing their susceptibility to bactericides and the host immune system, which makes biofilm formation the basis of many persistent infections in humans and animals (Mah, 2012). Bacteria growing in biofilms are often largely resistant to antimicrobial agents and the mechanisms of drug resistance of biofilm cells are not fully understood. In *A. pleuropneumoniae*, biofilm formation is prevalent among field isolates (Kaplan and Mulks, 2005). In addition, antimicrobial treatments were still necessary to prevent the spread of *A. pleuropneumoniae*, for that the currently available vaccination provides only partial protection and limited impact on morbidity (Bossé et al., 2015). Various antibiotic resistance patterns have been reported in *A. pleuropneumoniae*, which become a problem for the control of porcine pleuropneumonia outbreaks (Wang et al., 2010; Kucerova et al., 2011; Pridmore et al., 2011). Therefore, antibiotic resistance and biofilm formation present big challenges for antimicrobial therapies in *A. pleuropneumoniae*.

In *A. pleuropneumoniae*, several resistance genes or plasmids were identified (Wang et al., 2010; Archambault et al., 2012), while drug eﬄux pumps were poorly understood. In most Gram-negative bacteria, eﬄux pumps that actively extrude multiple structural classes of antibacterials out of the cells, play a key role in multidrug resistance (MDR) phenotypes (Blair et al., 2014). Eﬄux pumps were also suggested to be involved in antimicrobial resistance of biofilms and in the ability to form biofilms in several bacterial species: *Escherichia coli, Klebsiella pneumoniae*, and *Salmonella typhimurium* (Kvist et al., 2008; Van Acker and Coenye, 2016). Little is known about the contribution of eﬄux pumps to antibiotic resistance and biofilm formation in *A. pleuropneumoniae*.

Classically, eﬄux transporters can be divided into five families: ABC (ATP-binding cassette), RND (resistance nodulation division), MFS (major facilitator superfamily), SMR (small MDR), and MATE (multidrug and toxic compound extrusion) families or superfamilies (Li et al., 2015). Usually, the first three families of transporters function in the form of tripartite eﬄux pumps, which involve a periplasmic adaptor protein and an outer membrane eﬄux protein (OEP), which is usually TolC or a TolC-like homolog (Koronakis et al., 2004; Du et al., 2015). Clinically, the most significant eﬄux pumps in Gram-negative bacteria are members of the RND family (Morita et al., 2012; Anes et al., 2015), among which AcrAB-TolC of *E. coli* is best studied (Zgurskaya et al., 2011). With a channel-tunnel structure, TolC functions as an exit duct of AcrAB-TolC system for diverse types of substances when recruited by the substrate-engaged inner membrane complexes, conferring intrinsic resistance to a large variety of antimicrobial agents such as antibiotics, detergents as well as host-derived bile salts and antimicrobial peptides (Piddock, 2006; Horiyama et al., 2010). In fact, bacteria may have a number of different transporters, but only a limited number of OEPs, which makes TolC family proteins a key component of eﬄux pumps (Koronakis et al., 2004).

In this study, we identified two TolC-like proteins, encoded by the genes APL_RS01335 (*tolC1*) and APL_RS04400 (*tolC2*), respectively, in *A. pleuropneumoniae* genome. Analyses of the mutants revealed that *tolC*1, but not *tolC*2, was involved in MDR of planktonic and biofilm cells. In addition, *tolC*1 was also shown to be required for biofilm formation. Chemical inhibition of eﬄux pumps by Phe-Arg-β-naphthylamide (PAβN) not only increased the drug susceptibility, but also repressed biofilm formation and eradicated mature biofilms, indicating a potential application of eﬄux pump inhibitors (EPIs) as adjunctive therapies in antibacterial and antibiofilm strategies in *A. pleuropneumoniae*.

## Materials and Methods

### Strains and Growth Conditions

Bacterial strains, plasmids and primers used in this study are listed in **Tables 1** and **2**. Wild-type *A. pleuropneumoniae* strain SC1516, isolated from an outbreak on a farm in Sichuan, China in 2014, was used in the current study. SC1516 and derivatives were grown in Tryptic Soy Broth (TSB, Difco Laboratories, Detroit, MI, USA) or on Tryptic Soy agar (TSA, Difco Laboratories, Detroit, MI, USA) supplemented with 5% bovine serum (Invitrogen, USA) and 0.01% β-nicotinamide adenine dinucleotide (NAD). Where necessary, the media were supplemented with 50 μg/ml kanamycin or 5 μg/ml chloramphenicol. *E. coli* strains were grown on Luria-Bertani (LB, Difco Laboratories, Detroit, MI, USA) agar or in broth. When required, the media were supplemented with 25 μg/ml chloramphenicol and 1 mM diaminopimelic acid (Sigma-Aldrich, St. Louis, MO, USA). Unless otherwise stated, all strains were grown at 37°C.

**Table 1:** Bacterial strains and plasmids used in this study.

Strain or plasmid	Characteristics^a^	Source or reference
Strains		
*Actinobacillus pleuropneumoniae*		
SC1516	Serovar 7 clinical isolate	Laboratory collection
*tolC1*::*cat*	*tolC1* insertion mutant of SC1516	This study
Δ*tolC2*	*tolC2* deletion mutant of SC1516	This study
*tolC1*::*cat tolC1^+^*	The complemented strain of *tolC1::cat* containing the *tolC1* gene	This study
Δ*tolC2*/*tolC2*	The complemented strain of *ΔtolC2* containing the *tolC2* gene	This study
*Escherichia coli*		
DH5α	General cloning host strain	TAKARA
BL21 (DE3)	General expression host strain	TAKARA
β2155	thrB1004 pro thi hsdS lacZΔM15 (F′ lacZΔM15 lacI^q^ traD36 proA^+^ proB^+^) Δdap::erm (Erm^r^)	Baltes et al., 2003
Plasmids		
pMD19-T	Amp^r^, *E. coli* cloning vector	TAKARA
pET39b	Kan^r^, *E. coli* expression vector	Novagen
pEMOC2	Transconjugation vector based on pBluescript SK with mobRP4, a polycloning site, Cm^r^, and transcriptional fusion of the *omlA* promoter with the *sacB* gene	Baltes et al., 2003
pEMOC2-Δ*tolC2*	1000 bp left and 1000 bp right homologous fragment of *tolC2* cloned into pEMOC2	This study
pMC-express	Cm^r^, expression vector of *A. pleuropneumoniae*	Bosse et al., 2009
pLCT	pMD19-T carrying the 24–865 bp of *tolC1* gene fragment and an *cat* cassette	This study
pLS88	Broad-host-range shuttle vector from *Haemophilus ducreyi*; Str^r^ Kan^r^	Willson et al., 1989
pLStolC1	Kan^r^, *tolC1* gene with its native promoter in pLS88	This study
pLStolC2	Kan^r^, *tolC2* gene with its native promoter in pLS88	This study

*^a^Ermr, erythromycin resistance; Amp^r^, ampicillin resistance; Kan^r^, kanamycin resistance; Cm^r^, chloramphenicol resistance; Str^r^, streptomycin resistance; cat, chloramphenicol acetyltransferase.*

**Table 2:** Primers used in this study.

Primer name	Sequence (5′→3′)^a^	Description
*tolC1*-pET39b-F	AGTACTGACGGTTCGCTTGAACAAG (ScaI)	Primers for *tolC1* gene cloning and expression
*tolC1*-pET39b-R	CTCGAGTTATCTTCTATATTTACCC (XhoI)	
*tolC2*-pET39b-F	AGTACTTTGCAGTCCGATATTGAATTACC (ScaI)	Primers for *tolC2* gene cloning and expression
*tolC2*-pET39b-R	CTCGAGTTACCAACCGCCCCCAATCGAT(XhoI)	
*tolC2*-L-F	AGCGGGCCCGTCAAAAACGGTTCCGTTGC (ApaI)	Cloning the 1000 bp upstream of *tolC2* into pEMOC2
*tolC2*-L-R	AGTTGTTCCTATATTCTTAT	
*tolC2*-R-F	**ATAAGAATATAGGAACAACT**AATCTATACATTTCGAACGG	Cloning the 1000 bp downstream of *tolC2* into pEMOC2
*tolC2*-R-R	ATTTGCGGCCGCTAAATATTCCGCATAAACAC (NotI)	
*tolC1*-F	AACACTCTCCGTTGTACTTG	Primers used for cloning the 24–865 bp of *tolC1* gene
*tolC1*-R	**AATAAGGGTAACTATTGCCGC**ATCCGGGCGATTTGCGATTG	
cat-F	CGGCAATAGTTACCCTTATT	Primers used for amplification of the chloramphenicol resistance gene
cat-R	TTCAACTAACGGGGCAGGTT	
*tolC1*-pls88-F	CGGAATTCGCGTTTAAAACAAGCTTCTC (EcoRI)	Primers used for amplification of *tolC1* gene and its native promoter
*tolC1*-pls88-R	CATGCCATGGAGCGCAATACTATCTTCTAT (NcoI)	
*tolC2*-pls88-F	CGGAATTCCACAAAGTCTAACCCAACTT (EcoRI)	Primers used for amplification of *tolC2* gene and its native promoter
*tolC2*-pls88-R	CATGCCATGGTTATTGCTCCGTTCGAAATG (NcoI)	

*^a^Restriction sites are underlined. The 20-bp extensions required for overlap PCR are indicated in bold text.*

### Bioinformatic Analysis

The NCBI BLAST package was used to perform homology searches, and DNAMAN 4.0 was employed for similarity and identity analyses. A neighbor-joining tree containing 34 sequences from members of the TolC family was constructed using Mega 5.0 software program, and viewed through iTOL^1^.

### Construction of *tolC1* and *tolC2* Mutants and Genetically Complemented Strains in *A. pleuropneumoniae*

For creation of the *tolC1* mutant, a DNA fragment of 842-bp obtained from *tolC1* (from 24 to 865 bp) was joined with the chloramphenicol acetyl transferase gene (*cat*) from pMC-Express (Bosse et al., 2009) by overlap PCR. The resulting PCR product was cloned into a pMD19-T vector (Takara, Japan) to give pLCT. pLCT was electroporated into SC1516 and the transformants were selected on TSA supplemented with 5 μg/ml chloramphenicol, 5% bovine serum and 0.01% NAD, resulting in the *tolC1* mutant.

To delete the full length of *tolC2* gene, the 1000-bp upstream and downstream regions flanking the *tolC2* gene were PCR-amplified from chromosomal DNA of SC1516, and fused using overlap extension PCR. The resulting PCR product was cloned into the suicide vector pEMOC2 to generate plasmid pEMOC2-Δ*tolC2*. The resulting plasmid pEMOC2-Δ*tolC2* was conjugated into SC1516 using the *E. coli* strain β2155 (Xie et al., 2013). After two homologous recombination steps, the *tolC2* mutant was identified by PCR.

For genetic complementation, the entire coding regions of *tolC1* and *tolC2* together with their promoters were cloned into the shuttle vector pLS88 to generate plasmids pLS*tolC1* and pLS*tolC2*, respectively. The resulting plasmids were electroporated into the corresponding mutant strains and the transformants were selected on TSA with 5% bovine serum and 0.01% NAD containing kanamycin or kanamycin and chloramphenicol.

### Expression of Recombinant TolC1 and TolC2 and Generation of Rabbit Antisera

Recombinant proteins of TolC1 and TolC2 were generated using an *E. coli* expression system. Briefly, PCR fragments containing *tolC1* or *tolC2* genes minus their signal peptide sequences were amplified from genomic DNA of SC1516 using primers *tolC1*-pET39b-F/R and *tolC2*-pET39b-F/R, respectively. The resulting PCR products were cloned into the ScaI and XhoI sites of pET39b to form plasmid pET39b-TolC1 and pET39b-TolC2. These recombinant plasmids were transformed into *E. coli* BL21 (DE3) and induced to express by the addition of 0.5 mM isopropyl-beta-D-thiogalactopyranoside (IPTG). Recombinant proteins were purified by Ni affinity chromatography using Profinity IMAC Ni-Charged Resin (Bio-Rad) as described (Zhang et al., 2016).

Rabbit immunization was carried out in strict accordance with procedures approved by the Sichuan Agricultural University Institutional Animal Care and Use Committee. Antisera were generated by immunized each rabbit subcutaneously with 0.5 mg of recombinant TolC1 and TolC2 containing the same volume of complete Freund’s adjuvant (Sigma) on day 0 (primary immunization). On days 14 and 21, the rabbits were immunized subcutaneously with 0.5 mg of recombinant proteins that were mixed with incomplete Freund’s adjuvant (Sigma). Serum samples were collected 1 week after the final immunization and stored at -70°C.

### Western Blotting

Western blotting analyses were performed as described previously (Zhang et al., 2015). Briefly, whole-cell extracts of wild-type strain SC1516, mutants and genetically complemented strains were separated on 12% Sodium dodecylsulfate (SDS) – polyacrylamide gel electrophoresis (PAGE) and electrotransferred onto nitrocellulose membranes, respectively. After blocking in 5% skimmed milk in TBST (Tris-HCl buffered saline containing 0.05% Tween 20) at room temperature for 30 min, the membranes were incubated with rabbit antiserum for 3 h at room temperature. After three washes with TBST, the membranes were hybridized with horseradish peroxidase-conjugated goat anti-rabbit IgG antibody (Bioss, China) for 30 min at room temperature. Membranes were washed five times with TBST and the signal was detected with the Immun-Star Western C Kit (Bio-Rad, USA) according to the manufacturer’s instructions.

### *In vitro* Growth Assays

Growth of *A. pleuropneumoniae* SC1516 and its derivatives was determined by transferring 1 ml overnight culture into 100 ml of fresh TSB with 5% bovine serum and 0.01% NAD. Bacteria cultures were incubated while shaking at 37°C. Growth was monitored by measuring the optical density (OD) at 600 nm (OD_600_) with the SmartSpec Plus spectrophotometer (Bio-Rad).

### Antibiotic Susceptibility Assays

Assays on the minimal inhibitory concentration for planktonic cells (MIC-P) were carried out using 17 antibiotics, detergents and dyes that were reported to be eﬄux pump substrates (Martinez et al., 2009). The MIC-P was determined by a broth micro-dilution assay using serial twofold dilution in TSB with 5% bovine serum and 0.01% NAD. The microtiter plates were incubated at 37°C and visually scored for growth or no growth at 24 h. All antibiotics used in this study were obtained from Sigma-Aldrich, Co. (St. Louis, MO, USA). The experiments were independently performed at least three times in triplicate.

The minimal bactericidal concentrations for biofilms (MBC-B) were determined as described (Zhang and Mah, 2008). Overnight-grown biofilms in a 96-well microtiter plate were exposed to serially diluted antibiotics of choice for at least 12 h. Then, fresh TSB supplemented with 5% bovine serum and 0.01% NAD was used to replace the antibiotic-containing medium and incubated for additional 24 h. The viability of bacteria in biofilms was assessed by transferring a spot of the culture onto a TSA with 5% bovine serum and 0.01% NAD. The experiments were independently performed at least three times in triplicate.

For co-treatments of antibiotics and PAβN, appropriate final concentration (60 μg/ml for the MIC-P assay and 160 μg/ml for the MBC-B assay) of PAβN (Sigma, USA) was added into the medium containing antibiotics.

### Crystal Violet Biofilm Assays

Biofilm formation assays were performed as described previously with minor changes (Kaplan et al., 2004; Zhang et al., 2016). Briefly, overnight cultures of SC1516 and its derivatives were diluted 1/100 into fresh TSB with 5% bovine serum and 0.01% NAD and incubated statically in sterile 96-well microtiter plates (Costar 3599, Corning, NY, USA). After 12 h at 37°C, bacterial growth (OD_600_) was measured using a microplate reader (Bio-Rad iMark^TM^ microplate Reader). Culture supernatant was then removed and the wells were washed by immersing in water and stained with 100 μl of crystal violet (0.1%, w/v) per well for 5 min at room temperature. After that, crystal violet was removed and washed with distilled water. The bound dye was released by adding 100 μl of acetic acid (33%, v/v) and the optical density was measured at 595 nm using a microplate reader.

For the biofilm inhibition assays, overnight broth culture of SC1516 was diluted (1/100) into fresh TSB with 5% bovine serum and 0.01% NAD in the presence of PAβN (0, 20, 40, and 60 μg/ml) alone, or with subminimal inhibitory concentrations of antibiotic (0.25 μg/ml of ceftazidime, 0.0625 μg/ml of ofloxacin, or 1 μg/ml of gentamicin) for planktonic cells (sub-MIC-P). Control wells were without any antibiotics or PAβN. Biofilms quantified as described above.

For the eradication assays of established biofilms, overnight biofilms of SC1516 in a 96-well microtiter plate were exposed to PAβN (160 μg/ml), or subminimal-bactericidal concentration for biofilms (sub-MBC-B) of antibiotic (1 μg/ml of ceftazidime, 1 μg/ml of ofloxacin, or 10 μg/ml of gentamicin) with or without PAβN. Control wells were without any antibiotics or PAβN. After 24 h of incubation, supernatants were removed and replaced with fresh TSB with 5% bovine serum and 0.01% NAD for additional 12 h. Biofilms were stained with crystal violet and quantified as described above. All crystal violet biofilm assays were performed at least three times in triplicate.

### Confocal Laser Scanning Microscopy

Static biofilms were grown on 20-mm^2^ coverglasses submerged in 3 ml TSB with 5% bovine serum and 0.01% NAD in 6-well microtiter plate for 12 h. The supernatants were removed and the biofilms were exposed to 10 μg/ml of gentamicin with or without 160 μg/ml of PAβN for additional 24 h. Then biofilms were washed with water three times and stained with the LIVE/DEAD^@^ BacLight^TM^ Bacterial Viability Kit (catalog no. L13152; Molecular Probes, Invitrogen, USA) or Wheat Germ Agglutinin (WGA)–Oregon Green^@^ 488 (Invitrogen, Molecular Probes, Eugene, OR, USA). Plates were incubated for 15 min at room temperature in the dark and washed for three times with water. Stained biofilms were examined with a Nikon A1R confocal scanning laser microscope (CLSM). The images were analyzed using the NIS-Elements AR software (Nikon, Japan).

### Statistical Analysis

Data were analyzed with the GraphPad Prism version 6.0 (GraphPad Software, San Diego, CA, USA). One- or two-way analysis of variance (ANOVA) was employed to determine the difference between more than two groups. Differences were considered statistically significant at ^∗^*P* < 0.05.

## Results

### *A. pleuropneumoniae* Encodes Two TolC Homologs

Bioinformatic analysis showed the presence of two *tolC* homologs in the genomes of all serotypes of *A. pleuropneumoniae* available in NCBI. In strain SC1516, the two TolC homologs, named TolC1 and TolC2, are encoded by *tolC1* and *tolC2*, with GenBank accession numbers KU705812 and KU705813, respectively. TolC1 showed similarity to a number of bacterial TolC proteins detected through a BLAST search against the GenBank, including representative members from *Haemophilus influenzae* (HI1462; 56% identity), *Klebsiella pneumoniae* (CusC; 26% identity) and *E. coli* (TolC; 22% identity). TolC2 showed similarity to *Vibrio cholerae* VceC (30% identity), *Pseudomonas aeruginosa* OprM (27% identity) and *H. influenzae* HI1462 (23% identity). Besides, TolC1 and TolC2 proteins are both 464 amino acids with 99.78 and 98.98% sequence identity among different serotypes of *A. pleuropneumoniae*, respectively.

**FIGURE 1:**
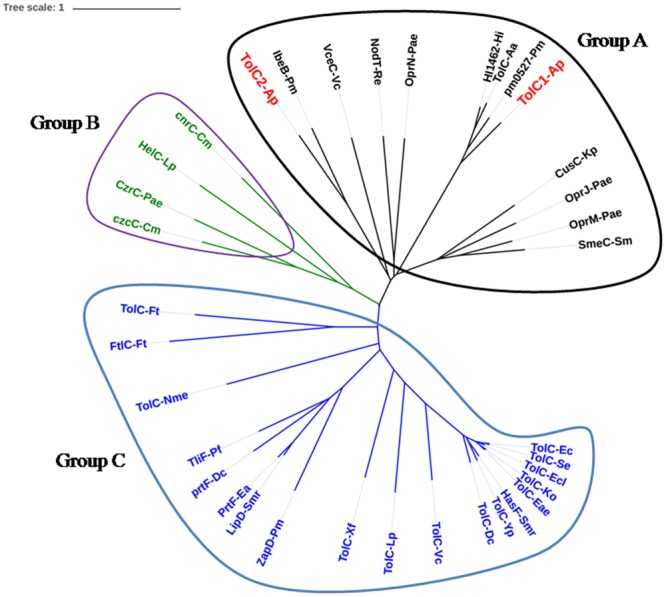
**Location of TolC1 and TolC2 of *Actinobacillus pleuropneumoniae* in the TolC family phylogenetic tree**. The TolC homologs can be grouped into three major clades, named Groups A, B, and C. Group A proteins seem to be mainly involved in multidrug eﬄux (black), Group B proteins mainly in cation eﬄux (green) and Group C proteins are multifunctional (blue). Proteins were labeled as “protein name-species name” according to details in Supplementary Table S1.

Phylogenetic analysis was performed to investigate the evolutionary relationships of *A. pleuropneumoniae* TolC1 and TolC2 to other members of the TolC family. Thirty six proteins (see Supplementary Table S1) were clustered into three different groups (groups A, B, and C; **Figure 1**). Both TolC1 and TolC2 of *A. pleuropneumoniae* were phylogenetically clustered into the group A that seems to be mainly involved in the multidrug eﬄux.

### Construction of *tolC1* and *tolC2* Mutants and Genetically Complemented Strains in *A. pleuropneumoniae*

Initially, we attempted to inactivate both *tolC1* and *tolC2* genes by the suicide vector pEMOC2 carrying a counter-selection system (Xie et al., 2013). A Δ*tolC2* was easily obtained while multiple attempts to delete *tolC1* were unsuccessful. Therefore, *tolC1* was disrupted using plasmid pLCT as described in Section “Materials and Methods.” Gene disruption in *tolC1*::*cat* and Δ*tolC2* mutants and genetic complementation in *tolC1*::*cat tolC1^+^* and Δ*tolC2*/*tolC2* strains were confirmed by PCR (Supplementary Figure S1).

**FIGURE 2:**
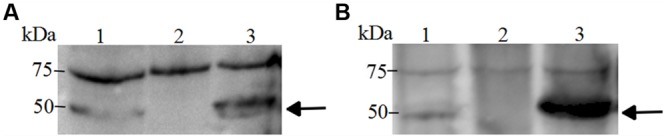
**Western blotting of TolC1 and TolC2 (A), Western blotting of TolC1 in whole-cell lysate of *A. pleuropneumoniae* probed with rabbit antiserum**. Lane 1, wild-type strain SC1516; Lane 2, *tolC1*::*cat* strain; Lane 3, genetically complemented *tolC1*::*cat tolC1^+^* strain. **(B)** Western blotting of TolC2 in whole-cell lysate of *A. pleuropneumoniae* probed with rabbit antiserum. Lane 1, wild-type strain SC1516; Lane 2, Δ*tolC2* strain; Lane 3, genetically complemented Δ*tolC2*/*tolC2* strain. Target proteins are indicated with an arrow. The bands at about 75 kDa showed a cross-reactive protein.

To further confirm the mutations, Western blotting was performed with rabbit antisera. We do not know how much cloned proteins were incorporated to the membrane fraction, but the mutation can be confirmed by reading the positive/negative band. For TolC1, the antiserum identified a 50 kDa protein in the wild-type strain SC1516 and in the genetically complemented strain *tolC1*::*cat tolC1^+^*, but absent in the *tolC1*::*cat* strain. Similarly, the antiserum against TolC2 detected a protein with the expected size in both SC1516 and genetically complemented Δ*tolC2*/*tolC2*, but not in Δ*tolC2* (**Figure 2**). The results were consistent with the PCR data, confirming that both *tolC1*::*cat* and Δ*tolC2* have lost their ability to express corresponding proteins. In contrast, the expression of TolC1 and TolC2 were restored in both genetically complemented mutants.

**FIGURE 3:**
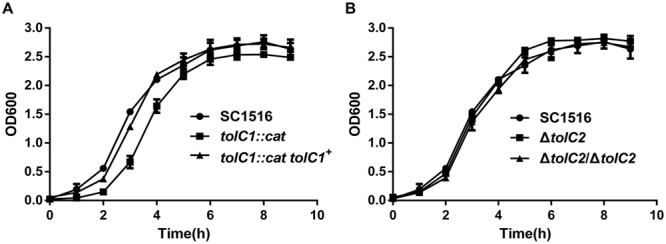
**Growth kinetics of the wild-type strain SC1516, *tolC* mutants and genetically complemented strains in TSB broth. (A)** The *tolC1* mutant showed a reduced growth rate in the log phage, while later reached similar optical density in the stationary phase. **(B)** The *tolC2* mutant exhibited the same growth kinetics with the wild-type strain. Data presented here are means of triplicate experiments and error bars represent standard deviations.

Next, we compared the growth of the wild-type SC1516 and its derivatives. The *tolC1*::*cat* strain showed a slight growth delay in the log phage, but reached similar optical density in the stationary phase (**Figure 3A**). Genetic complementation restored the growth kinetics of *tolC1*::*cat tolC1^+^* strain to the wild-type level. There was no significant difference in growth kinetics between SC1516 and Δ*tolC2* (**Figure 3B**).

### TolC1, but not TolC2, Is Part of the Multidrug Resistance Machinery of *A. pleuropneumoniae*

To determine the roles of TolC1 and TolC2 in MDR of planktonic cells, MIC-P assays were carried out using a broth micro-dilution assay. As shown in **Table 3**, for the *tolC1*::*cat* strain, the MICs of novobiocin, ceftazidime, and lincomycin decreased by 256-, 8- and 4-fold, while the MICs of nalidixic acid, kanamycin and tetracycline decreased by twofold, respectively. Additionally, *tolC1*::*cat* also showed enhanced susceptibility to various fluoroquinolones. Moreover, *tolC1*::*cat* was also more susceptible to non-antibiotic antimicrobials, including acriflavine, crystal violet and SDS by 8-, 8-, and 4-fold, respectively. In contrast, *tolC1*::*cat* showed similar levels of resistance to its parental wild-type SC1516 for gentamicin, rifampin and polymyxin B, suggesting that these compounds might not be substrates of TolC1-dependent eﬄux systems. Resistance to the majority of the compounds was returned back to wild-type levels in the genetically complemented strain *tolC1*::*cat tolC1^+^*, confirming that the observed antibiotic susceptibility of *tolC1*::*cat* mutant was not caused by polarity effects. In addition, no change was observed in sensitivity to vancomycin, suggesting that the loss of TolC1 seemed not to perturb outer membrane integrity of planktonic *A. pleuropneumoniae* negatively.

**Table 3:** Susceptibility of *A. pleuropneumoniae* strains to different antimicrobials.

Drugs	Test index^b^ (μg/ml)	Strains			
		SC1516	*tolC1*::*cat*	*tolC1*::*cat tolC1^+^*	SC1516+PAβN
Gentamicin	MIC-P	4	4	4	4
	MBC-B	40	10	40	20
Kanamycin	MIC-P	16	8	>32^a^	16
	MBC-B	160	160	/	/
Rifampin	MIC-P	0.5	0.5	0.5	0.015625
	MBC-B	8	2	8	2
Ceftazidime	MIC-P	1	0.125	1	0.5
	MBC-B	16	4	8	2
Novobiocin	MIC-P	16	0.0625	16	0.0625
	MBC-B	32	0.125	16	4
Ciprofloxacin	MIC-P	0.125	0.0625	0.125	0.125
	MBC-B	2	2	2	/
Naldixic acid	MIC-P	128	64	128	64
	MBC-B	320	80	320	160
Ofloxacin	MIC-P	0.25	0.125	0.25	0.125
	MBC-B	4	1	4	2
Enrofloxacin	MIC-P	0.25	0.0625	0.25	0.25
	MBC-B	2	2	2	/
Vancomycin	MIC-P	64	64	64	64
	MBC-B	640	640	640	640
Lincomycin	MIC-P	128	32	32	32
	MBC-B	160	40	80	/
Tetracycline	MIC-P	8	4	8	4
	MBC-B	32	16	32	/
Polymyxin B	MIC-P	2	2	2	1
	MBC-B	16	16	16	/
Deoxycholate	MIC-P	>400	100	>400	<100
	MBC-B	/	/	/	/
Acriflavine	MIC-P	1	0.125	1	0.5
	MBC-B	4	2	4	/
Crystal violet	MIC-P	8	1	4	1
	MBC-B	32	2	16	/
SDS	MIC-P	128	32	128	16
	MBC-B	160	40	160	/
PAβN	MIC-P	80	10	80	/
	MBC-B	320	80	160	/

*^a^The plasmids used in the complemented strains confer resistance to kanamycin. ^b^MIC-P, minimal inhibitory concentration for planktonic cells; MBC-B, the minimal bactericidal concentration for biofilms.*

Next, we used MBC-B assays to examine if TolC1 was involved in the antibiotic resistance of biofilm cells. The *tolC1*::*cat* biofilm bacteria had significantly increased sensitivity to varieties of antimicrobial agents (**Table 3**). In contrast, *tolC1*::*cat tolC1^+^* bacteria were as resistant as the wild-type SC1516. In general, TolC1 conferred resistance to same classes of antibiotics in both planktonic cells and biofilm cells, except for gentamicin and rifampin, to which the MBC-B of *tolC1*::*cat* mutant was both decreased by fourfold (**Table 3**). Interestingly, when compared against wild-type SC1516, Δ*tolC2* mutant did not show significant increase in susceptibility to any of these aforementioned antimicrobials (Supplementary Table S2). Collectively, these results suggested that TolC1 is involved in multidrug eﬄux and resistance in both planktonic and biofilm cells of *A. pleuropneumoniae* SC1516.

### The Mutant Lacking a Functional TolC Impairs Biofilm Formation in *A. pleuropneumoniae*

**FIGURE 4:**
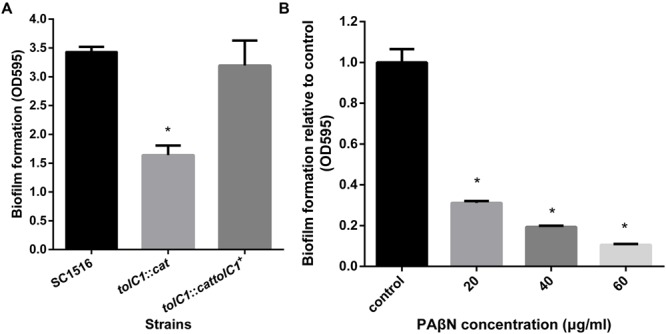
**The loss of TolC1 or the addition of PAβN impaired biofilm formation in *A. pleuropneumoniae*. (A)** The *tolC1*::*cat* had significantly reduced ability to form biofilms. **(B)** PAβN inhibited biofilm formation of wild-type strain SC1516 in a dose-dependent manner. Data indicate the mean of three independent experiments performed in duplicates and error bars show SDs. Asterisks indicate statistical significance using one-way ANOVA (^∗^*P* < 0.05).

Crystal violet biofilm assays were employed to examine the effect of *tolC1* mutation on biofilm formation of *A. pleuropneumoniae*. Biofilm phenotypes of wild-type strain SC1516, *tolC1*::*cat* mutant and the complemented strain *tolC1*::*cat tolC1^+^* were compared. The ability to form biofilms was decreased significantly in *tolC1*::*cat* (**Figure 4A**). In contrast, *tolC1*::*cat tolC1^+^* formed biofilms of wild-type level. Furthermore, SC1516, *tolC1*::*cat* and *tolC1*::*cat tolC1^+^* showed similar growth kinetics in static cultures (Supplementary Table S2A), which showed that the disruption of TolC1 did not cause a growth defect under the given culture conditions. The results indicated that inactivation of TolC1-dependent multidrug eﬄux pumps reduces the ability of *A. pleuropneumoniae* to form biofilms.

### PAβN Restores the Drug Susceptibility of Planktonic and Biofilm Cells in *A. pleuropneumoniae*

PAβN is a competitive inhibitor of RND eﬄux pumps (Zechini and Versace, 2009). We examined the susceptibility of wild-type SC1516 and *tolC1*::*cat* mutant to PAβN. The MIC of PAβN for *tolC1*::*cat* was eightfold lower than that for SC1516 and the genetically complemented *tolC1*::*cat tolC1^+^* (**Table 3**), indicating that PAβN was transported in a TolC1-dependent way in *A. pleuropneumoniae*. To determine the competitive inhibitory effect of PAβN on eﬄux pumps, novobiocin was employed as an indicator. Based on the MIC (80 μg/ml) of PAβN for wild-type SC1516, sub-MICs of PAβN (20, 40, and 60 μg/ml) were added for the novobiocin susceptibility assays. At 20 μg/ml, PAβN was insufficient to block drug eﬄux and the sensitivity to novobiocin was not affected. The novobiocin sensitivity was significantly increased 16-fold in the presence of 40 μg/ml of PAβN and 64-fold in the presence of 60 μg/ml of PAβN. Therefore, 60 μg/ml of PAβN was employed as the optimum concentration in subsequent MIC-P assays, and 160 μg/ml (1/2 MBC-B, **Table 3**) in the biofilm assays.

To determine the effect of PAβN on drug susceptibility of *A. pleuropneumoniae* planktonic cells, MIC-P assays were carried out in the presence of cocktail drugs composed of PAβN and antibiotics. PAβN increased the susceptibility of SC1516 to rifampin by 256-fold. Additionally, the susceptibility to ceftazidime, ofloxacin, lincomycin, and polymyxin B was increased by 2–4 fold. Moreover, MICs of acriflavine, deoxycholate, crystal violet, and SDS were decreased by 2–8 fold, while the MIC of novobiocin decreased by 64-fold (**Table 3**).

To assess the efficacy of PAβN on the antibiotic sensitivity of biofilm cells, MBC-B assays were performed with PAβN in a cocktail with novobiocin, ofloxacin, ceftazidime, nalidixic acid, gentamicin, and rifampin, respectively. Overnight biofilm cells showed 2–4 fold higher sensitivity to PAβN-antibiotic cocktails than individual antibiotic alone (**Table 3**). Taken together, these results indicate that PAβN efficiently inhibits multidrug eﬄux pumps and increases the killing activity of various classes of antibiotics against both planktonic and biofilm cells of *A. pleuropneumoniae*.

### PAβN Enhances the Inhibitory Effects of Antibiotics on Bacterial Biofilm Formation

Crystal violet staining assay was also employed to assess the effect of PAβN on biofilm formation. The addition of PAβN significantly reduced biofilm formation of SC1516 in a dose-dependent manner (**Figure 4B**). The effective concentrations of PAβN required were below the MIC (80 μg/ml), indicating that PAβN performed a specific antibiofilm effect rather than a substantial inhibition on planktonic growth (Supplementary Figure S2B).

**FIGURE 5:**
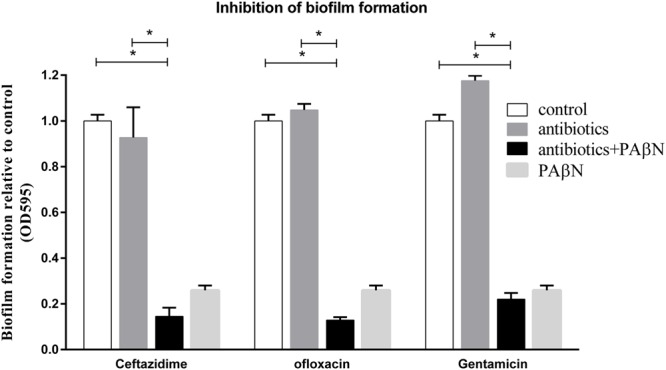
**Inhibition of biofilm formation of *A. pleuropneumoniae* by antibiotics with or without the EPI PAβN**. Biofilm formation of the WT strain SC1516 was significantly inhibited by subinhibitory concentrations of antibiotics (0.25 μg/ml of ceftazidime, 0.0625 μg/ml of ofloxacin or 1 μg/ml of gentamicin) in combination with PAβN (60 μg/ml). Asterisks indicate statistical significance using two-way ANOVA (^∗^*P* < 0.05).

Because PAβN increased drug susceptibility of biofilm cells and suppressed biofilm formation, we examined if the antibiofilm efficacies of sub-MIC-P of antibiotics (ceftazidime, ofloxacin, or gentamicin) could be enhanced by PAβN. Low concentration of antibiotic alone caused no significant inhibition, and in some cases, or even induced biofilm formation (**Figure 5**). Importantly, when compared against individual antibiotics alone, addition of PAβN significantly enhanced the ability of these antimicrobials to inhibit biofilm formation by the wild-type strain SC1516 (**Figure 5**). Furthermore, cocktails of PAβN with ceftazidime or ofloxacin were slightly more effective than PAβN alone in inhibiting biofilm formation.

### PAβN Enhances the Eradication Effects of Antibiotics on Established Biofilms

**FIGURE 6:**
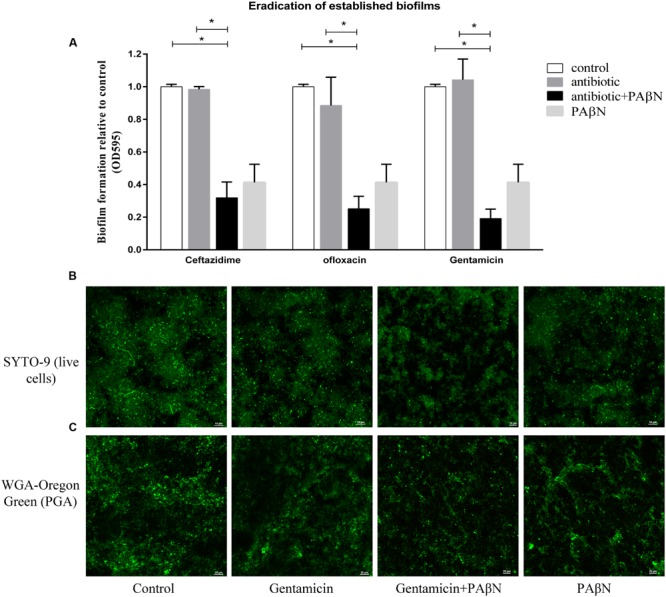
**Eradication of established biofilms of *A. pleuropneumoniae* by antibiotics with or without PAβN. (A)** Biofilm eradication was significantly increased by sub-bactericidal concentrations of antibiotics (1 μg/ml of ceftazidime, 1 μg/ml of ofloxacin, or 10 μg/ml of gentamicin) in combination with PAβN (160 μg/ml). Data indicate mean of three independent experiments performed in duplicates, error bars indicate SD, ^∗^*P* < 0.05 when compared to the untreated control using two-way ANOVA. CLSM images of persistent biofilm cells with intact cell membranes were colored green by SYTO 9 **(B)** and the biofilm matrix was characterized by staining PGA with WGA-Oregon green **(C)**. Overnight grown biofilms of *A. pleuropneumoniae* SC1516 were significantly eradicated by gentamicin combining PAβN, showing sparse bacteria colonies and scattered WGA fluorescent spots.

Given its significant inhibitory effect on biofilm formation, the cocktail PAβN-antibiotic strategy might be efficacious in the eradication on established biofilms. Biofilm eradication assays were performed using sub-MBC-B of ceftazidime, ofloxacin, and gentamicin plus PAβN. As shown in **Figure 6A**, low concentration of each antibiotic alone had no effect on biofilm eradication, while the combination of each antibiotic with PAβN diminished the mature biofilms significantly. These results indicated that the eradication activity of sub-MBC-B of antibiotics on biofilms was substantially enhanced by PAβN.

To visualize the eradication of mature biofilms by cocktail of PAβN-antibiotics, CLSM assays were performed as described in Section “Materials and Methods.” Biofilms were stained with SYTO-9 and propidium iodide to label live and dead bacteria, respectively. Biofilm architectures were characterized using a WGA fluorescent probe that specifically labeled exopolysaccharide PGA, the key scaffolding component of *A. pleuropneumoniae* biofilms (Hathroubi et al., 2016). Biofilm eradication was determined by the relative abundance of live cells and the content of PGA. When compared against the untreated group, the addition of gentamicin alone did not diminish pre-established biofilms. In contrast, a cocktail drug composed of gentamicin with PAβN substantially eradicated biofilms (**Figures 6B,C**). Both viable cells and biofilm matrix were reduced, showing sparse bacteria colonies and scattered WGA fluorescent spots. Taken together, these data demonstrate that PAβN enhances the ability of various antibiotics to efficiently inhibit biofilm formation as well as diminish established biofilms in *A. pleuropneumoniae*.

## Discussion

The bacterial TolC protein is multi-functional, and the most distinguished role is to function as part of multidrug eﬄux system that contributes to bacterial MDR (Zgurskaya et al., 2011). In this study, we show that *A. pleuropneumoniae* encodes TolC1 and TolC2, which are highly conserved among all serotypes of *A. pleuropneumoniae*. The presence of multiple TolC family proteins is common in Gram-negative bacteria (Gil et al., 2006; Kamal and Shafer, 2010). Generally, inactivation of any one of those TolC-like proteins would alter antimicrobial susceptibility to some extent (van Amsterdam et al., 2005; Lee et al., 2014). Interestingly, our study demonstrates that only TolC1 forms a part of the MDR machinery of *A. pleuropneumoniae* (**Table 3**; Supplementary Table S2), even though both TolC1 and TolC2 are phylogenetically clustered as members of the multidrug eﬄux group (**Figure 1**). In our work, we tried to construct a double mutant and after many attempts, we failed due to an unsolved reason. The actual role of TolC2 needs further study. A recent study speculated that TolC2 may function in conjunction with the MacAB-like proteins to mediate the biofilm dispersal in *A. pleuropneumoniae* (Tremblay et al., 2013).

So, which proteins encoded by the *A. pleuropneumoniae* genome could provide additional components for multidrug eﬄux in association with TolC1 to pump antibacterial compounds? Till now, no eﬄux pump has been characterized in *A. pleuropneumoniae* yet. Genome analysis of the serotype 5b reference strain in GenBank predicts five multidrug eﬄux pumps: an RND pump encoded by APL_0587 (*acrB*); an ABC family transporter encoded by APL_0626 (*macB*); a MATE family eﬄux transporter encoded by APL_0369 (*norM*); a SMR-type transporter encoded by APL_1607 (*emrE*) and a major facilitator superfamily pump encoded by APL_0355 (*bcr*). Additional studied pumps include the membrane fusion protein AcrA encoded by APL_0586 and the MacA homolog by APL_1814. Among them, AcrAB would most likely to work with TolC1, allowing for the eﬄux of a broad range of toxic compounds, as revealed in other pathogens (Li and Nikaido, 2009).

To date, most published studies on TolC-dependent eﬄux pumps have focused its function on MDR for planktonic cells, with little known for biofilm cells (Anes et al., 2015). Biofilm-dwelling bacteria are well-known to be more tolerant to the antimicrobials than planktonic cells. However, the resistance mechanisms are still not fully understood. Biofilm cells of the *tolC1* mutant strain showed significantly increased drug susceptibility when compared to that of the parental strain, indicating that TolC1 forms part of the multidrug eﬄux machinery of *A. pleuropneumoniae* that mediate intrinsic MDR of both planktonic and biofilm cells.

Unexpectedly, biofilm cells of the *tolC1*::*cat* mutant were more susceptible to gentamicin and rifampin than that of the parental strain, which showed no change in MIC-P assays (**Table 3**). It is possible that changes in the composition of biofilm matrix of *tolC1*::*cat* mutant caused the increased sensitivity to gentamicin and rifampin (Mah, 2012). Additionally, inactivation of TolC1 was confirmed to impair biofilm formation. Therefore, the ability to form competent biofilms appears to be another important function of TolC1 in contribution to MDR of biofilm cells of *A. pleuropneumoniae.*

Bacterial eﬄux pumps, especially the clinically relevant AcrAB-TolC, are often considered as important targets for developing novel antibacterial treatments (Blair et al., 2014). Consequently, EPIs, especially those against RND-type pumps, attracted a lot of attention as potential adjunctive therapies that would improve the potency of antibiotics and decrease the emergence of MDR bacteria (Opperman and Nguyen, 2015). The EPI PAβN is known as a competitive inhibitor of RND eﬄux pumps (Kinana et al., 2016). In this study, the increased susceptibility of *tolC1::cat* to PAβN suggested a role for TolC1 in PAβN eﬄux. Given the predicted eﬄux pumps in *A. pleuropneumoniae*, PAβN is most likely to target AcrB and competitively inhibit AcrAB-TolC system. Our results showed that PAβN inhibited the eﬄux pumps of *A. pleuropneumoniae* in a dose-dependent manner, with 60 μg/ml the most effective against planktonic bacteria and 160 μg/ml against the biofilm cells. Significantly, this concentration contrasted with that in a previous study, in which 20 μg/ml of PAβN was sufficient to increase drug susceptibility from 77 to 96% for *E. coli* MG1655 (Sáenz et al., 2004). This discrepancy suggested that the effects of PAβN may vary to different bacterial species (Lister et al., 2012).

PAβN has been used as a classical EPI of RND pumps, but due to the dicationic character, its membrane-permeabilizing effect, especially when used at high concentrations, has frequently been ignored (Li et al., 2015). The OM-permeabilizing effect could also increase antibiotic susceptibility, which is likely to interfere with the judgment of PAβN on eﬄux pump inhibition. In this study, when combined with 60 μg/ml PAβN in MIC-P assays, the MIC of vancomycin—an antibiotic that is unable to cross the outer membrane of Gram-negative bacteria—was not changed (**Table 3**). These data suggested that 60 μg/ml of PAβN seemed not to cause substantial outer membrane damage of *A. pleuropneumoniae*. When 160 μg/ml of PAβN was used in biofilm cells, the susceptibility to vancomycin was also not changed with or without PAβN (**Table 3**). Besides, the effect of PAβN on drug susceptibility of biofilm cells were also determined in the presence of 1 mM MgSO4 to stabilize the outer membrane, as described previously (Lamers et al., 2013). As a result, the supplementation with magnesium did not restore the resistance to gentamicin, rifampin, ceftazidime, novobiocin, nalidixic acid, ofloxacin (data not shown), indicating that the ability of PAβN to increase the drug susceptibility of biofilm cells seemed not due to its permeabilizing activity.

Bacteria are capable of establishing biofilms on almost any surfaces. Therefore, prevention of biofilm formation is the key in thwarting biofilm-related infections. In clinics, bacteria are often exposed to low concentration of antibiotics at the beginning and end of a drug regimen, or continuously during low-dose therapy (Kaplan, 2011). Several studies, including ours, have shown that low doses of some antimicrobials induce biofilm formation in a variety of bacterial species (**Figure 5**; Hoffman et al., 2005; Kaplan et al., 2012). Importantly, our results indicate that PAβN not only effectively suppresses the induction of biofilm formation by low doses of antibiotics, but also significantly enhances the therapeutic potential of low doses of antibiotics in inhibiting biofilm formation of *A. pleuropneumoniae*.

Bacterial cells within established biofilms are highly resistant to external antimicrobial agents (Mah, 2012). Because eﬄux pumps confer many types of bacterial drug resistance (Blair et al., 2014), not surprisingly, interference with the eﬄux pump activity of biofilm cells restores the antibiotic sensitivity of biofilms. Therefore, cocktail drugs composed of EPI with antibiotics are projected to have greater potential of destroying established biofilms. Indeed, PAβN significantly increases the ability of sub-bactericidal concentration of antibiotics to eradicate established biofilms of *A. pleuropneumoniae*. The demonstration that PAβN suppresses biofilm formation whilst also potentiates the eradication of established biofilms implies important therapeutic value of the EPI.

## Conclusion

We have characterized two TolC-like proteins, TolC1 and TolC2, and only TolC1 function as a component of the MDR machinery of *A. pleuropneumoniae.* TolC1-dependent eﬄux pump(s) contributed to drug resistance of cells in planktonic culture as well as in biofilms. Moreover, we demonstrated a biofilm defect for the mutant lacking TolC1, which facilitating an important role of TolC1 in biofilm formation of *A. pleuropneumoniae*. Chemical inhibiting eﬄux pumps by PAβN improved the efficacy of antibiotics as well as repressed biofilm formation of *A. pleuropneumoniae*. In addition, cocktail drugs composed of EPI with low concentration of antibiotics, showed great potential to eradicate biofilms, which is important in treating chronic infectious diseases. These findings may contribute to understanding the molecular basis of MDR of *A. pleuropneumoniae*, especially for the drug-tolerance biofilm cells, and representing a step in controlling MDR and biofilm-related infections in *A. pleuropneumoniae.*

## Author Contributions

SC, XW, and YW designed the experiments. XH, RW, QY, and QZ performed the experiments with assistance of YH. YL and LZ analyzed the data and wrote the paper, GL edited the manuscript. All authors read, commented on and approved the final manuscript.

## Conflict of Interest Statement

The authors declare that the research was conducted in the absence of any commercial or financial relationships that could be construed as a potential conflict of interest.

## Footnotes

1 http://itol.embl.de/
