# Supplementary material for: A TolC-Like Protein of Actinobacillus pleuropneumoniae Is Involved in Antibiotic Resistance and Biofilm Formation
Source: Front Microbiol. 2016 Oct 24;7:1618. doi: 10.3389/fmicb.2016.01618 (PMC5075564; doi:10.3389/fmicb.2016.01618)
Supplement: Supplementary file 1 [file Data_Sheet_1.DOCX]

**One of two TolC-like proteins is involved in antibiotic resistance and biofilm formation of *Actinobacillus pleuropneumoniae* clinical isolate SC1516**

**Supplemental information**


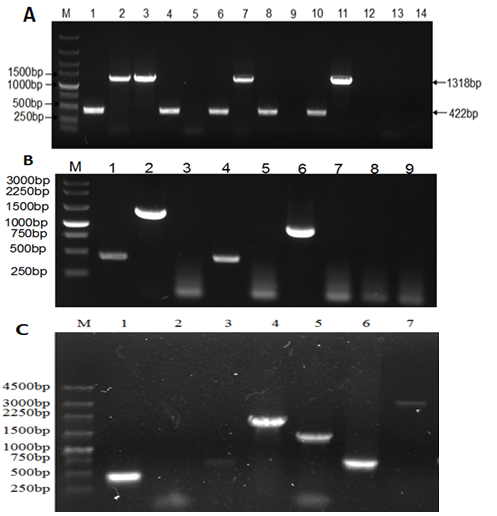


**FIGURE** **S1| PCR analysis of *tolC1* and *tolC2* mutation. (A) Confirmation of *tolC* mutation and genetic complementation.** PCR analysis of genomic DNA extracted from wild type strain SC1516 (lanes 1, 2, 3) for positive control, *tolC1*::*cat* (lanes 4, 5), *tolC1*::*cat tolC1^+^* (lanes 6, 7), Δ*tolC2* (lanes 8, 9) and Δ*tolC2*/*tolC2* (lanes 10, 11) using primers APXIVA-1L/R ([Schaller et al., 2001](#_ENREF_25)) (lanes1, 4, 6, 8, 10); *tolC1*-pET39b-F/R (lanes 2, 5, 7) and *tolC2*-pET39b-F/R (lanes 3, 9, 11). Lane M: DNA marker DL5000 (Takara, Dalian, China); Lanes 12, 13, 14: negative controls using primer pairs APXIVA-1L/R, *tolC1*-pET39b-F/R and *tolC2*-pET39b-F/R, respectively. Primers APXIVA-1L/R were used specifically for the identification of *A. pleuropneumoniae* strains. **(B) Confirmation of *tolC1* mutation.** PCR analysis of genomic DNA extracted from wild type strain SC1516 (lanes 1, 2, 3) for positive control and *tolC1*::*cat* (lanes 4, 5, 6) using primers APXIVA-1L/R (lanes 1 and 4), *tolC1*-pET39b-F/R (lanes 2 and 5) and cat-F/R (lanes 3 and 6), respectively. Lane M: DNA marker 250bp ladder (Takara, Dalian, China); Lanes 7, 8, 9: negative controls using primer pairs APXIVA-1L/R, *tolC1*-pET39b-F/R and cat-F/R, respectively. The results from lanes 2 and lane 5 showed that the *tolC1* gene was disrupted in the *tolC1*::*cat* genome. The results from lane 3 and lane 6 showed that the *tolC1*::*cat* strain was marked with chloramphenicol resistance. **(C) Confirmation of *tolC2* mutation.** PCR analysis of genomic DNA extracted from Δ*tolC2* using primers APXIVA-1L/R (lane 1), *tolC*2-pET39b-F/R (lane 2), Cm-F/R (lane 3) and TolC2-L-F/ TolC2-R-R (lane 4), respectively. lanes 5, 6 and 7: positive controls amplified from genomic DNA of SC1516 using primer pairs *tolC2*-pET39b-F/R, Cm-F/R and TolC2-L-F/TolC2-R-R, respectively. The primers Cm-F/R was used to amplify the chloramphenicol resistance gene of pEMOC2: Cm-F：5’ -TTTCAGGAGCTAAGGAAG- 3’; Cm-R：5’ -CACCAATAACTG CCTTAA- 3’. Lane M: DNA marker 250bp ladder (Takara, Dalian, China); The results of lane 2 and lane 5 showed that the *tolC2* was deleted from the Δ*tolC2* genome. The results of lane 4 (2000bp) and lane 7 (3394bp) showed that the *tolC2* was deleted and only the upstream and downstream regions flanking the *tolC2* gene existed. The result of lane 2 showed the *tolC2* mutant was actually markerless.


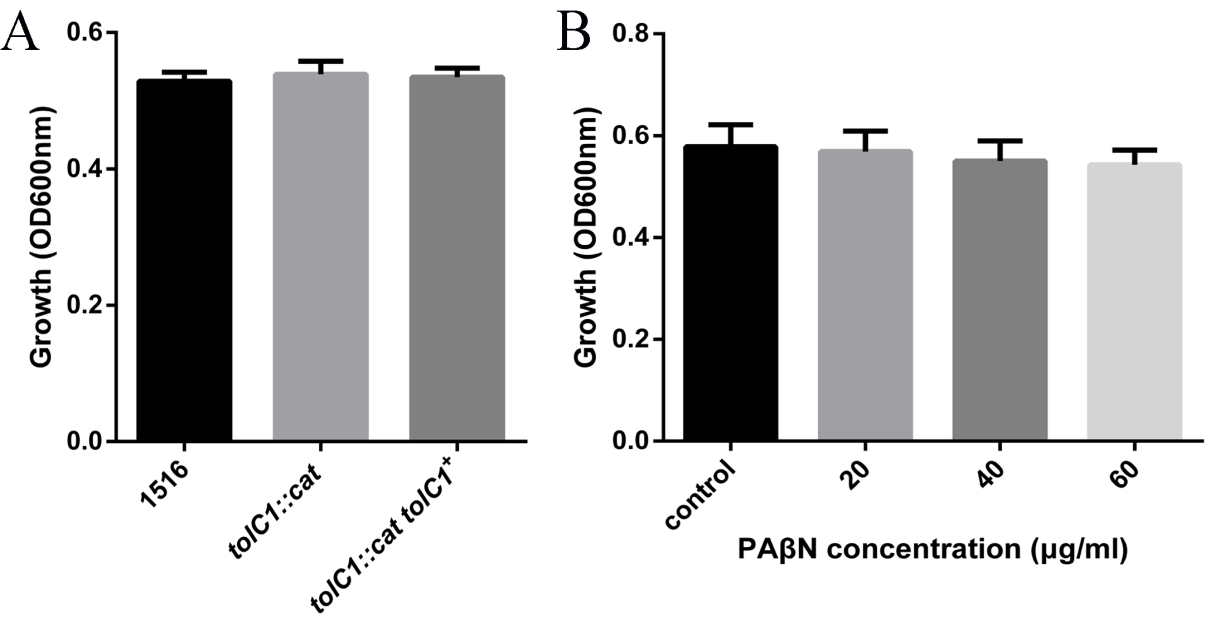


**FIGURE S2| Comparison of bacterial growth during biofilm assays.** (A), no difference in growth was observed among three *A. pleuropneumoniae* strains after 12 h of static culture during biofilm formation assays. (B), Subinhibitory concentrations of PAβN did not inhibit the growth of the wild-type strain SC1516.

**TABLE S1 Homologues used in the phylogenetic tree analysis of *A. pleuropneumoniae* TolC proteins**

| Name | Organism | NCBI accession no. or GI no. | Reference |
| --- | --- | --- | --- |
| TolC1-AP  HI1462-Hi  TolC-Aa  Pm0527-Pm  NodT-Re  OprN-Pae  TolC2-AP  IbeB-Pm  OprJ-Pae  OprM-Pae  SmeC-Sm  HelC-Lp  CusC-Kp  CnrC-Cm  CzrC-Pae  CzcC-Cm  VceC-Vc  TolC-Nme  PrtF-Eam  LipD-Smr  TliF-Pf  PrtF-Dc  ZapD-Pmi  TolC-Ft  FtlC-Ft  TolC-Xf  TolC-Lp  TolC-Vc  TolC-Dc  TolC-Yp  HasF-Smr  TolC-Se TolC-Ec  TolC-Ecl  TolC-Eae  TolC-Ko | *Actinobacillus pleuropneumoniae*  *Haemophilus influenza*  *Actinobacillus actinomycetemcomitans*  *Pasteurella multocida*  *Rhizobium etli*  *Pseudomonas aeruginosa*  *Actinobacillus pleuropneumoniae*  *Pasteurella multocida*  *Pseudomonas aeruginosa*  *Pseudomonas aeruginosa*  *Stenotrophomonas maltophilia*  *Legionella pneumophila*  *Klebsiella pneumoniae*  *Cupriavidus metallidurans*  *Pseudomonas aeruginosa*  *Cupriavidus metallidurans*  *Vibrio Cholerae*  *Neisseria meningitides*  *Erwinia amylovora*  *Serratia marcescens*  *Pseudomonas fluorescens*  *Dickeya chrysanthemi*  *Proteus mirabilis*  *Francisella tularensis*  *Francisella tularensis*  *Xylella fastidiosa*  *Legionella pneumophila*  *Vibrio cholera*  *Dickeya chrysanthemi*  *Yersinia pestis*  *Serratia marcescens*  *Salmonella enterica*  *Escherichia coli*  *Enterobacter cloacae*  *Enterobacter aerogenes*  *Klebsiella oxytoca* | KU705812  gi\|16273365\|  gi\|122536995\|  gi\|12720790\|  gi\|86359005\|  gi\|15597691  KU705813  gi\|12722419\|  gi\|116052739  gi\|3184190\|  gi\|5764626\|  gi\|54298314\|  gi\|597517340\|  gi\|521152\|  gi\|5921516  gi\|1731916\|  gi\|61680586\|  gi\|6900447\|  gi\|4826418\|  gi\|3080540\|  gi\|4063019\|  gi\|41550\|  gi\|3493599\|  gi\|88954016\|  gi\|88954014\|  gi\|71899951\|  gi\|53753515\|  gi\|11135318\|  gi\|15987119\|  gi\|908266008\|  gi\|48773763\|  gi\|799383\|  gi\|90111528\|  gi\|112182851\|  gi\|17384364\|  gi\|111433457\| | This study  ([Trepod and Mott, 2004](#_ENREF_26))  ([Crosby and Kachlany, 2007](#_ENREF_7))  ([Hatfaludi et al., 2008](#_ENREF_14))  ([Cosme et al., 2008](#_ENREF_6))  ([Linares et al., 2005](#_ENREF_17))  This study  ([Hatfaludi et al., 2008](#_ENREF_14))  ([Masuda et al., 2000b](#_ENREF_21))  ([Masuda et al., 2000a](#_ENREF_20))  ([Cosme et al., 2008](#_ENREF_6))  ([Ferhat et al., 2009](#_ENREF_10))  ([Zulfiqar and Shakoori, 2012](#_ENREF_30))  ([Grass et al., 2000](#_ENREF_12))  ([Hassan et al., 1999](#_ENREF_13))  ([Nies, 1992](#_ENREF_22))  ([Federici et al., 2005](#_ENREF_8))  ([Kamal et al., 2007](#_ENREF_15))  ([Zhang et al., 1999](#_ENREF_29))  ([Akatsuka et al., 1995](#_ENREF_2))  ([Ahn et al., 1999](#_ENREF_1))  ([Letoffe et al., 1990](#_ENREF_16))  ([Wassif et al., 1995](#_ENREF_27))  ([Gil et al., 2006](#_ENREF_11))  ([Gil et al., 2006](#_ENREF_11))  ([Reddy et al., 2007](#_ENREF_24))  ([Ferhat et al., 2009](#_ENREF_10))  ([Bina and Mekalanos, 2001](#_ENREF_4))  ([Letoffe et al., 1990](#_ENREF_16))  ([Lister et al., 2012](#_ENREF_18))  ([Begic and Worobec, 2008](#_ENREF_3))  ([Buckley et al., 2006](#_ENREF_5))  ([Zgurskaya et al., 2011](#_ENREF_28))  ([Perez et al., 2012](#_ENREF_23))  ([Masi et al., 2007](#_ENREF_19))  ([Fenosa et al., 2009](#_ENREF_9)) |

**TABLE S2 Susceptibility of *A. pleuropneumoniae* SC1516, Δ*tolC2* and genetically- complemented Δ*tolC2/tolC2* strain to different antimicrobials**

| Drug class | Compounds | MIC (μg/ml) for: | | | | |
| --- | --- | --- | --- | --- | --- | --- |
|  |  | SC1516 | Δ*tolC2* | | Δ*tolC2/tolC2* | |
| Aminoglycoside | Gentamicin | 4 | 8 | | 8 | |
|  | Kanamycin | 16 | 16 | >32^b^ | |  |
| Amphenicol | Chloramphenicol | 0.5 | 0.5 | | 0.5 | |
|  | rifampin | 0.5 | 0.5 | | 0.5 | |
| Beta-lactam | Ampicillin | 8 | 8 | | 32 | |
|  | Ceftazidime | 1 | 4 | | 4 | |
|  | cephalosporin | 1 | 1 | | 0.5 | |
| Coumarin | Novobiocin | 16 | 16 | | 8 | |
| Fluoroquinolone | Ciprofloxacin | 0.125 | 0.0625 | | 0.125 | |
|  | Norfloxacin | 0.5 | 0.5 | | 0.5 | |
|  | Ofloxacin | 0.25 | 0.25 | | 0.25 | |
| Quinolones | Enrofloxacin  Naldixic acid | 0.25  128 | 0.25  128 | | 0.25  128 | |
| Glycopeptide | Vancomycin | 64 | 64 | | 32 | |
| Lincosamide | Lincomycin | 128 | 128 | | 64 | |
| Tetracyclines | Tetracycline | 8 | 16 | | 16 | |
| polypeptide | Polymyxin B | 2 | 4 | | 4 | |
| Bile salts | Deoxycholate | >400 | >400 | | >400 | |
| Dyes | Acriflavine | 1 | 1 | | 1 | |
|  | Crystal violet | 8 | 8 | | 8 | |
| Surfactants | SDS | 128 | 128 | | 128 | |

**References**

Ahn, J.H., Pan, J.G., and Rhee, J.S. (1999). Identification of the tliDEF ABC transporter specific for lipase in *Pseudomonas fluorescens* SIK W1. *J Bacteriol* 181**,** 1847-1852.

Akatsuka, H., Kawai, E., Omori, K., and Shibatani, T. (1995). The three genes *lipB*, *lipC*, and *lipD* involved in the extracellular secretion of the *Serratia marcescens* lipase which lacks an N-terminal signal peptide. *J Bacteriol* 177**,** 6381-6389.

Begic, S., and Worobec, E.A. (2008). The role of the *Serratia marcescens* SdeAB multidrug efflux pump and TolC homologue in fluoroquinolone resistance studied via gene-knockout mutagenesis. *Microbiology* 154**,** 454-461.

Bina, J.E., and Mekalanos, J.J. (2001). *Vibrio cholerae* *tolC* is required for bile resistance and colonization. *Infect Immun* 69**,** 4681-4685.

Buckley, A.M., Webber, M.A., Cooles, S., Randall, L.P., La Ragione, R.M., Woodward, M.J., and Piddock, L.J. (2006). The AcrAB-TolC efflux system of *Salmonella enterica serovar Typhimurium* plays a role in pathogenesis. *Cell Microbiol* 8**,** 847-856.

Cosme, A.M., Becker, A., Santos, M.R., Sharypova, L.A., Santos, P.M., and Moreira, L.M. (2008). The outer membrane protein TolC from *Sinorhizobium meliloti* affects protein secretion, polysaccharide biosynthesis, antimicrobial resistance, and symbiosis. *Mol Plant Microbe Interact* 21**,** 947-957.

Crosby, J.A., and Kachlany, S.C. (2007). TdeA, a TolC-like protein required for toxin and drug export in *Aggregatibacter (Actinobacillus) actinomycetemcomitans*. *Gene* 388**,** 83-92.

Federici, L., Du, D., Walas, F., Matsumura, H., Fernandez-Recio, J., Mckeegan, K.S., Borges-Walmsley, M.I., Luisi, B.F., and Walmsley, A.R. (2005). The crystal structure of the outer membrane protein VceC from the bacterial pathogen *Vibrio cholerae* at 1.8 A resolution. *J Biol Chem* 280**,** 15307-15314.

Fenosa, A., Fuste, E., Ruiz, L., Veiga-Crespo, P., Vinuesa, T., Guallar, V., Villa, T.G., and Vinas, M. (2009). Role of TolC in *Klebsiella oxytoca* resistance to antibiotics. *J Antimicrob Chemother* 63**,** 668-674.

Ferhat, M., Atlan, D., Vianney, A., Lazzaroni, J.C., Doublet, P., and Gilbert, C. (2009). The TolC protein of *Legionella pneumophila* plays a major role in multi-drug resistance and the early steps of host invasion. *PLoS One* 4**,** e7732.

Gil, H., Platz, G.J., Forestal, C.A., Monfett, M., Bakshi, C.S., Sellati, T.J., Furie, M.B., Benach, J.L., and Thanassi, D.G. (2006). Deletion of TolC orthologs in *Francisella tularensis* identifies roles in multidrug resistance and virulence. *Proc Natl Acad Sci U S A* 103**,** 12897-12902.

Grass, G., Grosse, C., and Nies, D.H. (2000). Regulation of the cnr cobalt and nickel resistance determinant from *Ralstonia sp.* strain CH34. *J Bacteriol* 182**,** 1390-1398.

Hassan, M.T., Van Der Lelie, D., Springael, D., Romling, U., Ahmed, N., and Mergeay, M. (1999). Identification of a gene cluster, czr, involved in cadmium and zinc resistance in *Pseudomonas aeruginosa*. *Gene* 238**,** 417-425.

Hatfaludi, T., Al-Hasani, K., Dunstone, M., Boyce, J., and Adler, B. (2008). Characterization of TolC efflux pump proteins from *Pasteurella multocida*. *Antimicrob Agents Chemother* 52**,** 4166-4171.

Kamal, N., Rouquette-Loughlin, C., and Shafer, W.M. (2007). The TolC-like protein of *neisseria meningitidis* is required for extracellular production of the repeats-in-toxin toxin FrpC but not for resistance to antimicrobials recognized by the Mtr efflux pump system. *Infect Immun* 75**,** 6008-6012.

Letoffe, S., Delepelaire, P., and Wandersman, C. (1990). Protease secretion by *Erwinia chrysanthemi*: the specific secretion functions are analogous to those of *Escherichia coli* alpha-haemolysin. *EMBO J* 9**,** 1375-1382.

Linares, J.F., Lopez, J.A., Camafeita, E., Albar, J.P., Rojo, F., and Martinez, J.L. (2005). Overexpression of the multidrug efflux pumps MexCD-OprJ and MexEF-OprN is associated with a reduction of type III secretion in *Pseudomonas aeruginosa*. *J Bacteriol* 187**,** 1384-1391.

Lister, I.M., Raftery, C., Mecsas, J., and Levy, S.B. (2012). *Yersinia pestis* AcrAB-TolC in antibiotic resistance and virulence. *Antimicrob Agents Chemother* 56**,** 1120-1123.

Masi, M., Saint, N., Molle, G., and Pages, J.M. (2007). The *Enterobacter aerogenes* outer membrane efflux proteins TolC and EefC have different channel properties. *Biochim Biophys Acta* 1768**,** 2559-2567.

Masuda, N., Sakagawa, E., Ohya, S., Gotoh, N., Tsujimoto, H., and Nishino, T. (2000a). Contribution of the MexX-MexY-oprM efflux system to intrinsic resistance in *Pseudomonas aeruginosa*. *Antimicrob Agents Chemother* 44**,** 2242-2246.

Masuda, N., Sakagawa, E., Ohya, S., Gotoh, N., Tsujimoto, H., and Nishino, T. (2000b). Substrate specificities of MexAB-OprM, MexCD-OprJ, and MexXY-oprM efflux pumps in *Pseudomonas aeruginosa*. *Antimicrob Agents Chemother* 44**,** 3322-3327.

Nies, D.H. (1992). CzcR and CzcD, gene products affecting regulation of resistance to cobalt, zinc, and cadmium (czc system) in *Alcaligenes eutrophus*. *J Bacteriol* 174**,** 8102-8110.

Perez, A., Poza, M., Fernandez, A., Fernandez Mdel, C., Mallo, S., Merino, M., Rumbo-Feal, S., Cabral, M.P., and Bou, G. (2012). Involvement of the AcrAB-TolC efflux pump in the resistance, fitness, and virulence of *Enterobacter cloacae*. *Antimicrob Agents Chemother* 56**,** 2084-2090.

Reddy, J.D., Reddy, S.L., Hopkins, D.L., and Gabriel, D.W. (2007). TolC is required for pathogenicity of *Xylella fastidiosa* in Vitis vinifera grapevines. *Molecular plant-microbe interactions* 20**,** 403-410.

Schaller, A., Djordjevic, S.P., Eamens, G.J., Forbes, W.A., Kuhn, R., Kuhnert, P., Gottschalk, M., Nicolet, J., and Frey, J. (2001). Identification and detection of *Actinobacillus pleuropneumoniae* by PCR based on the gene apxIVA. *Vet Microbiol* 79**,** 47-62.

Trepod, C.M., and Mott, J.E. (2004). Identification of the *Haemophilus influenzae tolC* gene by susceptibility profiles of insertionally inactivated efflux pump mutants. *Antimicrob Agents Chemother* 48**,** 1416-1418.

Wassif, C., Cheek, D., and Belas, R. (1995). Molecular analysis of a metalloprotease from *Proteus mirabilis*. *J Bacteriol* 177**,** 5790-5798.

Zgurskaya, H.I., Krishnamoorthy, G., Ntreh, A., and Lu, S. (2011). Mechanism and Function of the Outer Membrane Channel TolC in Multidrug Resistance and Physiology of Enterobacteria. *Front Microbiol* 2**,** 189.

Zhang, Y., Bak, D.D., Heid, H., and Geider, K. (1999). Molecular characterization of a protease secreted by *Erwinia amylovora*. *J Mol Biol* 289**,** 1239-1251.

Zulfiqar, S., and Shakoori, A.R. (2012). Molecular characterization, metal uptake and copper induced transcriptional activation of efflux determinants in copper resistant isolates of *Klebsiella pneumoniae*. *Gene* 510**,** 32-38.
